# Hypersensitivity reactions to anticancer agents: Data mining of the public version of the FDA adverse event reporting system, AERS

**DOI:** 10.1186/1756-9966-30-93

**Published:** 2011-10-05

**Authors:** Kaori Kadoyama, Akiko Kuwahara, Motohiro Yamamori, Brown, Toshiyuki Sakaeda, Yasushi Okuno

**Affiliations:** 1 Graduate School of Pharmaceutical Sciences, Kyoto University, Kyoto 606-8501, Japan; 2 School of Pharmacy and Pharmaceutical Sciences, Mukogawa Women's University, Nishinomiya 663-8179, Japan; 3 Kyoto Constella Technologies Co Ltd., Kyoto 604-8156, Japan

## Abstract

**Background:**

Previously, adverse event reports (AERs) submitted to the US Food and Drug Administration (FDA) database were reviewed to confirm platinum agent-associated hypersensitivity reactions. The present study was performed to confirm whether the database could suggest the hypersensitivity reactions caused by anticancer agents, paclitaxel, docetaxel, procarbazine, asparaginase, teniposide, and etoposide.

**Methods:**

After a revision of arbitrary drug names and the deletion of duplicated submissions, AERs involving candidate agents were analyzed. The National Cancer Institute Common Terminology Criteria for Adverse Events version 4.0 was applied to evaluate the susceptibility to hypersensitivity reactions, and standardized official pharmacovigilance tools were used for quantitative detection of signals, i.e., drug-associated adverse events, including the proportional reporting ratio, the reporting odds ratio, the information component given by a Bayesian confidence propagation neural network, and the empirical Bayes geometric mean.

**Results:**

Based on 1,644,220 AERs from 2004 to 2009, the signals were detected for paclitaxel-associated mild, severe, and lethal hypersensitivity reactions, and docetaxel-associated lethal reactions. However, the total number of adverse events occurring with procarbazine, asparaginase, teniposide, or etoposide was not large enough to detect signals.

**Conclusions:**

The FDA's adverse event reporting system, AERS, and the data mining methods used herein are useful for confirming drug-associated adverse events, but the number of co-occurrences is an important factor in signal detection.

## Background

Hypersensitivity reactions (HSRs), though rare in response to anticancer agents, are caused by certain classes of agents including platinum agents (cisplatin, carboplatin, and oxaliplatin), taxanes (paclitaxel and docetaxel), procarbazine and asparaginase, and epipodophyllotoxins (teniposide and etoposide) [1-5]. Despite comparatively lower frequency, doxorubicin and 6-mercaptopurine are also recognized as infrequent contributors to HSRs, and additionally other agents, e.g., 5-fluorouracil, cyclophosphamide and cytarabine, are thought to be agents that can potentially result in HSRs [1,3]. The use of the term "hypersensitivity" is widely used in clinical reports, though its use is also sporadic, and no exact definition is provided. It includes a wide array of symptoms from mild flushing and itching to lethal anaphylaxis. The pathogenic mechanisms by which the reactions occur are still unclear, although they seem to vary widely among agents. The exact prevalence of these reactions is difficult to evaluate, and such a problems is hindering the establishment of treatments.

Previously, pharmacoepidemiological studies have been conducted to confirm that adverse events have accompanied the use of cisplatin, carboplatin, and oxaliplatin [6,7]. More than a million case reports on adverse events (AERs) submitted to the US Food and Drug Administration (FDA) database were used, and a statistically significant association with an adverse event was detected as a signal, by applying standardized official pharmacovigilance methods [8-14]. This database relies on reports of spontaneous adverse events to the FDA generated by health professionals, consumers, and manufacturers, and the system is referred to as the Adverse Event Reporting System (AERS). These platinum agents have been proven to cause nausea, vomiting, acute renal failure, neutropenia, thrombocytopenia, and peripheral sensory neuropathy [6]. In terms of susceptibility, their rank-order was consistent with clinical observations, suggesting the usefulness of the AERS database and the data mining method used [6]. The National Cancer Institute Common Terminology Criteria for Adverse Events (NCI-CTCAE) version 4.0 was applied to evaluate the susceptibility to hypersensitivity reactions, and carboplatin and oxaliplatin were proved to cause mild, severe, or lethal reactions [7]. However, the same analytical method failed to detect signals for cisplatin-associated reactions [7]. In the present study, AERs submitted to the FDA were analyzed to detect signals for HSRs caused by paclitaxel, docetaxel, procarbazine, asparaginase, teniposide, and etoposide, in order to more clarify the critical factors to reproduce the clinical observations on HSRs. Additionally, agents thought to be associated with HSRs were also analyzed, including doxorubicin, 6-mercaptopurine, 5-fluorouracil, cyclophosphamide and cytarabine.

## Methods

### Data sources

Input data for this study were taken from the public release of the FDA's AERS database, which covers the period from the first quarter of 2004 through the end of 2009. The data structure of AERS is in compliance with international safety reporting guidance, ICH E2B, consisting of 7 data sets; patient demographic and administrative information (DEMO), drug/biologic information (DRUG), adverse events (REAC), patient outcomes (OUTC), report sources (RPSR), drug therapy start and end dates (THER), and indications for use/diagnosis (INDI). The adverse events in REAC are coded using preferred terms (PTs) in the Medical Dictionary for Regulatory Activities (MedDRA) terminology.

Prior to analysis, all drug names were unified into generic names by a text-mining approach, because AERS permits the registering of arbitrary drug names, including trade names and abbreviations. Spelling errors were detected by GNU Aspell and carefully confirmed by working pharmacists. Foods, beverages, treatments (e.g. X-ray radiation), and unspecified names (e.g., beta-blockers) were omitted for this study. Duplicated reports were deleted according to FDA's recommendation of adopting the most recent CASE number, resulting in the reduction of the number of AERs from 2,231,029 to 1,644,220. The primary and secondary suspected drugs were subjected to investigation as well as concomitant drugs.

### Definition of adverse events

According to the NCI-CTCAE version 4.0, AERs with PT10020751/hypersensitivity in REAC were adopted as the reports on mild HSRs, in which 19 lower level terms (LLTs) were assigned in MedDRA version13.0, including LLT10000656/acute allergic reaction, LLT10001718/allergic reaction, LLT10020756/hypersensitivity reaction, LLT10020759/hypersensitivity symptom, LLT10038195/red neck syndrome, and LLT10046305/upper respiratory tract hypersensitivity reaction (site unspecified). AERs with PT10011906/death (with 13 LLTs) or death terms in OUTC were excluded for mild HSRs. AERs with PT10002198/anaphylactic reaction were adopted as the reports on severe HSRs, in which 13 LLTs were assigned, including LLT10000663/acute anaphylactic reaction and LLT10002218/anaphylaxis. AERs both with PT10020751/hypersensitivity, and with PT10011906/death or death terms in OUTC were adopted as the reports on lethal HSRs. Of note, LLT10001718/allergic reaction and LLT10002218/anaphylaxis are also respectively assigned as allergic reactions and anaphylaxis in the NCI-CTCAE version 4.0, and PTs in their higher levels were used in this study.

### Data mining

In pharmacovigilance analysis, data mining algorithms have been developed to identify drug-associated adverse events as signals that are reported more frequently than expected by estimating expected reporting frequencies on the basis of information on all drugs and all events in the database [12-14]. For example, the proportional reporting ratio (PRR) [8], the reporting odds ratio (ROR) [9], the information component (IC) [10], and the empirical Bayes geometric mean (EBGM) [11] are widely used, and indeed, the PRR is currently used by the Medicines and Healthcare products Regulatory Agency (MHRA), UK, the ROR by the Netherlands Pharmacovigilance Centre, the IC by the World Health Organization (WHO), and the EBGM by the FDA.

All of these algorithms extract decision rules for signal detection and/or calculate scores to measure the associations between drugs and adverse events from a two-by-two frequency table of counts that involve the presence or absence of a particular drug and a particular event occurring in case reports. These algorithms, however, differ from one another in that the PRR and ROR are frequentist (non-Bayesian), whereas the IC and EBGM are Bayesian. In this section, only the scoring thresholds used in the present study are given, and the reader is referred to review articles for more extensive details of each statistical test [12-14].

Here, we define how a drug and associated adverse event is classified as a signal when using each statistical test. Using the PRR, a drug-event pair is classified as a signal if the event count ≥ 3 and the PRR ≥ 2.0 with an associated χ^2 ^value ≥ 4.0 [8]. Using the ROR, a signal is detected if the lower bound of the 95% two-sided confidence interval (CI) exceeds 1 [9]. Signal detection using the IC is done using the IC025 metric, a criterion indicating the lower bound of the 95% two-sided CI of the IC, and a signal is detected with the IC025 value exceeds 0 [10]. Finally, the EB05 metric, a lower one-sided 95% confidence limit of EBGM [11], is used and a signal is detected when EB05 is greater than or equal to the threshold value 2.0.

## Results

Table 1 lists the total number of adverse events occurring with each anticancer agent we investigated, and therein the numbers of co-occurrences with mild, severe or lethal HSRs. The total number of adverse events was less than 10,000 for procarbazine, asparaginase, teniposide, and 6-mercaptopurine, and those occurring with HSRs did not exceed 30 in total per agent. For etoposide and cytarabine, about 30,000 adverse events were found in total, but the number of HSRs co-occurrences counted was only about 50.

**Table 1:** The number of adverse events occurring with each anticancer agent

	N ^a)^	Mild ^b)^	Severe ^b)^	Lethal ^b)^
paclitaxel	42,038	228 *	79 *	12 *
docetaxel	36,983	79	18	17 *
procarbazine	1,287	1	0	0
asparaginase	6,414	1	5	2
teniposide	151	1	0	0
etoposide	28,264	31	25	3
doxorubicin	47,834	101	41	9
6-mercaptopurine	9,170	17	13	0
5-fluorouracil	40,282	108 *	44	10 *
cyclophosphamide	70,728	110	51	9
cytarabine	31,765	20	24	3

a) the total number of adverse events occurring with each anticancer agent.

b) the number of co-occurrences of mild, severe and lethal hypersensitivity reactions.

*: A signal was detected by at least 1 of 4 statistical indices

The statistical data on 5 other agents, paclitaxel, docetaxel, doxorubicin, 5-fluorouracil, and cyclophospamide, are summarized in Tables 2, 3 and 4. As shown in Table 2, the signals were detected for paclitaxel- and 5-fluorouracil-associated mild HSRs with 228 and 108 co-occurrences, respectively, but the association was only marginal for the latter. No signals were detected for docetaxel, doxorubicin, and cyclophospamide. As for severe reaction, the signal was detected for paclitaxel, but no signals for other four (Table 3). The associations with lethal reactions were detected for paclitaxel, docetaxel and 5-fluorouracil (Table 4).

**Table 2:** Signal detection for anticancer agent-associated mild hypersensitivity reactions

	N	PRR (χ2)	ROR (95% two-sided CI)	IC (95% two-sided CI)	EBGM (95% one-sided CI)
paclitaxel	228	2.768 * (254.855)	2.788 * (2.438, 3.117)	1.450 * (1.262, 1.638)	2.707 * (2.425)
					
docetaxel	79	1.087 (0.463)	1.087 (0.871, 1.302)	0.109 (-0.209, 0.427)	1.073 (0.890)
					
doxorubicin	101	1.074 (0.445)	1.074 (0.884, 1.265)	0.095 (-0.187, 0.376)	1.064 (0.902)
					
5-fluorouracil	108	1.365 (10.154)	1.366 * (1.130, 1.601)	0.436 * (0.164, 0.708)	1.344 (1.145)
					
cyclophosphamide	110	0.791 (5.894)	0.790 (0.655, 0.925)	-0.342 (-0.612, -0.073)	0.788 (0.673)
					

The total number of co-occurrences with mild hypersensitivity reactions was 43,288.

N: the number of co-occurrences of each anticancer agent out of 43,288 pairs, PRR: the proportional reporting ratio, ROR: the reporting odds ratio, IC: the information component, EBGM: the empirical Bayes geometric mean.

*: signal detected, see "Methods" for the detection criteria.

**Table 3:** Signal detection for anticancer agent-associated severe hypersensitivity reactions

	N	PRR (χ2)	ROR (95% two-sided CI)	IC (95% two-sided CI)	EBGM (95% one-sided CI)
paclitaxel	79	2.273 * (55.041)	2.278 * (1.826, 2.730)	1.151 * (0.833, 1.469)	2.174 (1.803)
					
docetaxel	18	0.588 (4.805)	0.587 (0.370, 0.805)	-0.773 (-1.431, -0.115)	0.591 (0.401)
					
doxorubicin	41	1.036 (0.021)	1.036 (0.762, 1.309)	0.032 (-0.408, 0.471)	1.014 (0.782)
					
5-fluorouracil	44	1.320 (3.102)	1.321 (0.982, 1.659)	0.374 (-0.051, 0.799)	1.276 (0.994)
					
cyclophosphamide	51	0.871 (0.851)	0.871 (0.661, 1.080)	-0.209 (-0.604, 0.185)	0.862 (0.683)
					

The total number of co-occurrences with severe hypersensitivity reactions was 18,255.

N: the number of co-occurrences of each anticancer agent out of 18,255 pairs, PRR: the proportional reporting ratio, ROR: the reporting odds ratio, IC: the information component, EBGM: the empirical Bayes geometric mean.

*: signal detected, see "Methods" for the detection criteria.

**Table 4:** Signal detection for anticancer agent-associated lethal hypersensitivity reactions

	N	PRR (χ2)	ROR (95% two-sided CI)	IC (95% two-sided CI)	EBGM (95% one-sided CI)
paclitaxel	12	2.623 * (10.495)	2.631 * (1.492, 3.770)	1.165 * (0.363, 1.967)	1.992 (1.237)
					
docetaxel	17	4.224 * (38.715)	4.247 * (2.635, 5.858)	1.800 * (1.121, 2.478)	3.268 * (2.062)
					
doxorubicin	9	1.728 (2.086)	1.731 (0.900, 2.563)	0.614 (-0.305, 1.533)	1.401 (0.819)
					
5-fluorouracil	10	2.281 * (5.977)	2.286 * (1.228, 3.344)	0.964 * (0.089, 1.838)	1.735 (1.037)
					
cyclophosphamide	9	1.169 (0.083)	1.170 (0.608, 1.731)	0.127 (-0.792, 1.046)	1.047 (0.613)

The total number of co-occurrences with lethal hypersensitivity reactions was 2,397.

N: the number of co-occurrences of each anticancer agent out of 2,397 pairs, PRR: the proportional reporting ratio, ROR: the reporting odds ratio, IC: the information component, EBGM: the empirical Bayes geometric mean.

*: signal detected, see “Methods” for the detection criteria.

## Discussion

The AERS database covers several million case reports on adverse events. Pharmacovigilance analysis aims to search for previously unknown patterns and automatically detect important signals, i.e., drug-associated adverse events, from such a large database. Recently developed data mining tools for pharmacovigilance have been successful at detecting signals that could not be found by individual case reviews and that warrant further investigation together with continuous surveillance. For this reason, data mining tools are being routinely used for pharmacovigilance, supporting signal detection and decision-making at companies, regulatory agencies, and pharmacovigilance centers [8-14]. Despite some limitations inherent to spontaneous reporting, the AERS database is a rich resource and the data mining tools provide a powerful means of identifying potential associations between drugs and adverse events.

Although HSRs are considered uncommon during treatment with anticancer agents, platinum agents, taxanes, procarbazine, asparaginase, and epipodophyllotoxins are thought to increase the susceptibility to such reactions [1-5]. Previously [7], and in this study, pharmacoepidemiological analyses were performed to confirm the HSRs caused by these agents, using more than a million AERs submitted to the FDA. The NCI-CTCAE version 4.0 was applied to evaluate the susceptibility to HSRs. Carboplatin, oxaliplatin, and paclitaxel were statistically demonstrated to be associated with mild, severe, and lethal HSRs, and docetaxel was associated with lethal reactions. No signals were detected for cisplatin, procarbazine, asparaginase, teniposide, and etoposide. For these latter agents, the total number of co-occurrences with HSRs was less than 100. Although the application of the NCI-CTCAE version 4.0 might have the effect on reproducibility of clinical observations, the total number of adverse events occurring with each anticancer agent we investigated and the number of co-occurrences of HSRs would be important factors.

In this study, we tried to evaluate the demographic effect on the susceptibility to severe HSRs. The ratio of male/female/unknown was 22/49/8 for the patients with paclitaxel-related severe HSR and the average value of age was 57.4 ± 15.0 years. These values were not different from those for all AERs. Similarly to paclitaxel, we could not figure out the effects of gender or age, in the cases of docetaxel and 5-fluorouracil. Additionally, the total number of drugs co-administered with 5-fluorouracil was 211 in 44 co-occurrences, and 29 of 211 was oxaliplatin, which is a well-established cause of HSRs. The co-administration drugs also can be confounding factor, and further analysis should be done with much larger numbers of co-occurrences.

Taxanes show poor water solubility, and are formulated with low molecular weight surfactants, for example, Cremophor EL and Tween 80 (polysorbate 80). These surfactants might contribute to HSRs. Although it is still controversial whether the surfactants or taxane moiety is responsible for HSRs [3,4,15-17], the difference between paclitaxel and docetaxel with regard to susceptibility might be explained by the surfactants [3,4]. Recently, surfactant-free novel derivatives and formulations have been developed. Their safety profiles will shed light on the debate about taxane-associated HSRs.

5-Fluorouracil, generally, is considered to be rarely associated with HSRs, although there are scattered reports of anaphylactic reactions occurring during or after its intravenous administration [18-21]. However, in this analysis, signals were detected for mild and lethal HSRs, and the susceptibility was comparable with that of docetaxel (Tables 2 and 4). This might be explained by co-administered oxaliplatin as stated. 5-Fluorouracil is used for cutaneous diseases such as psoriasis and actinic keratoses, and an irritant contact dermatitis is frequently seen [22-25]. This might be counted as hypersensitivity. Furthermore, hand-foot syndrome, a major adverse event of 5-fluorouracil, is characterized by painful erythematous lesions which mainly affect palmoplantar surfaces [26-28]. This syndrome might affect to analysis, because professionals could easily recognize symptoms involving sweat-associated toxicity, which is not a HSR, yet non-professionals might be mislead to classify the symptom as a HSR.

## Conclusions

AERs submitted to the FDA were analyzed using statistical techniques to establish the anticancer agent-associated HSRs. Based on 1,644,220 AERs from 2004 to 2009, the signals were detected for paclitaxel-associated mild, severe, and lethal HSRs, and docetaxel-associated lethal reactions. However, the total number of adverse events occurring with procarbazine, asparaginase, teniposide, or etoposide was not large enough to detect signals. The database and the data mining methods used herein are useful, but the number of co-occurrences is an important factor in signal detection.

## Competing interests

The author declares that they have no competing interests.

## Authors' contributions

KK, AK, MY, and TS made conception, designed and coordinated the study. YO and JB carried out calculations and statistical analysis. KK, JB and TS prepared the manuscript. All authors read and approved the final manuscript.
